# Polysaccharide Aldehydes and Ketones: Synthesis and Reactivity

**DOI:** 10.1021/acs.biomac.4c00020

**Published:** 2024-03-15

**Authors:** Zhenghao Zhai, Kevin J. Edgar

**Affiliations:** † Macromolecules Innovation Institute, Virginia Tech, Blacksburg, Virginia 24061, United States; ‡ Department of Sustainable Biomaterials, Virginia Tech, Blacksburg, Virginia 24061, United States

## Abstract

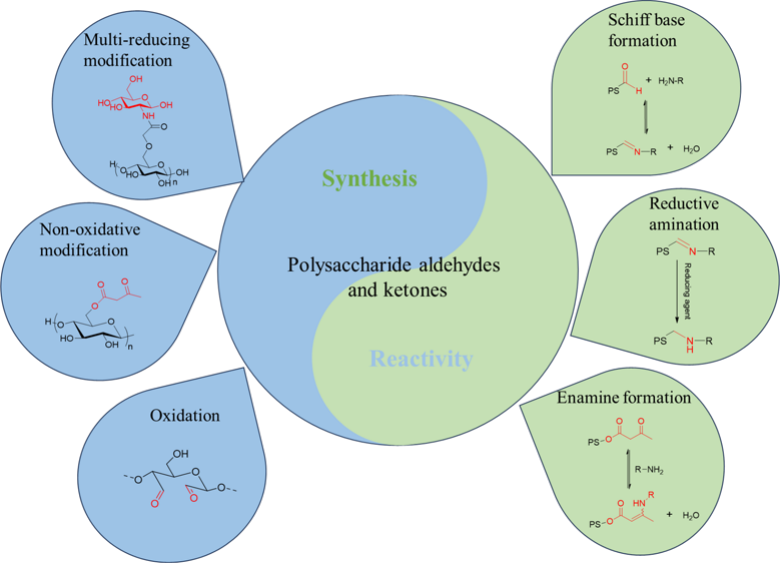

Polysaccharides are biodegradable, abundant, sustainable, and often benign natural polymers. The achievement of selective modification of polysaccharides is important for targeting specific properties and structures and will benefit future development of highly functional, sustainable materials. The synthesis of polysaccharides containing aldehyde or ketone moieties is a promising tool for achieving this goal because of the rich chemistry of aldehyde or ketone groups, including Schiff base formation, nucleophilic addition, and reductive amination. The obtained polysaccharide aldehydes or ketones themselves have rich potential for making useful materials, such as self-healing hydrogels, polysaccharide–protein therapeutic conjugates, or drug delivery vehicles. Herein, we review recent advances in synthesizing polysaccharides containing aldehyde or ketone moieties and briefly introduce their reactivity and corresponding applications.

## 1 Introduction

Polysaccharides are important members of the family of natural polymers and more chemically complex than other important families, such as proteins and poly(nucleic acids). They are abundant, diverse, typically benign, and always biodegradable. Despite their numerous advantages, natural polysaccharides have properties that may, in some cases, limit their ability to meet material demands of human society. For example, cellulose has a very strong tendency to self-associate (and crystallize) due in part to the formation of hydrogen bonding networks, which makes it completely insoluble in water or in any single organic solvent.^1,2^ The poor solubility of cellulose, coupled with its lack of observable glass-transition or melting temperatures, makes it difficult to process, thus impeding applications, such as packaging.^3^ This has inspired chemists to develop ways to chemically modify polysaccharides to enhance processability and adapt their structures to achieve desired performance.^4−6^ However, many of these methods lack regioselectivity. Regioselective modification of polysaccharides is challenging because they all contain multiple chemically nonequivalent alcohols, which nonetheless have similar reactivity; in some cases, polysaccharides also contain other reactive groups (e.g., carboxyl, amine, amide groups). These characteristics complicate the site-specific chemical modification of polysaccharides. Thus, most published polysaccharide modification reactions lead to relatively random substitution, which impedes a deeper understanding of structure–property relationships, targeting of desired properties, and optimal design of sustainable materials.

Aldehydes or ketones are valuable substituents because they can undergo reactions distinct from those of the numerous polysaccharide hydroxy groups. Important examples include the ability of aldehydes (or ketones) to react with amines to form imines or to be reductively aminated to form amines.^7−9^ The useful reactions available to aldehydes (or ketones) can enable site-specific chemical modification of polysaccharides. Unfortunately, all natural polysaccharides have only one aldehyde group per chain (at the reducing end); therefore, only one substituent can be attached per molecule in this way.^10−13^ To obtain polysaccharide derivatives by regioselective introduction of aldehydes or ketones, reliable chemical methods are needed to introduce an adequate degree of substitution (DS) of these functional groups.

This review summarizes previous synthetic methods for making polysaccharides with aldehyde or ketone substituents and briefly introduces their applications. We wish to help the reader choose, optimize, and probe different synthetic strategies for preparing polysaccharide aldehydes or ketones.

## 2 Synthetic Methods

In this section, we divide the synthetic strategies into three categories: oxidation, nonoxidative derivatization, and multireducing end modifications. Definitions, methods, functional groups introduced, and key features are summarized in Table 1. The detailed experimental parameters, green aspects, yields, and maximum DS values or conversions are summarized in Table 2.

**Table 1:** Strategies and Methods for the Synthesis of Polysaccharide Aldehydes and Ketones

strategy	definitions	methods	functional groups introduced	key features
oxidation	oxidize polysaccharide (PS) hydroxy groups to afford aldehydes or ketones	periodate oxidation	aldehydes	• well-established
				• vicinal diols are cleaved to afford aldehydes
				• PS chains degraded
		bleach oxidation	ketones	• secondary hydroxy groups on oligo(hydroxypropyl) chain termini oxidized to afford ketones
				• controllable degradation of PS chains
				• need for hydroxypropyl substituents
nonoxidative modification	react with small molecules to introduce aldehydes or ketones	esterification with 4-formylbenzoates	aldehydes	• esterification of PS with 4-formylbenzoic acid
				• 4-formylbenzoic acid may have safety issues.
		acetoacetylation	ketones	• highly reactive
				• limited thermal stability
		esterification with levulinate groups	ketones	• improved thermal stability
				• side reactions may occur
multireducing end modification	attach monosaccharides to PS	glucosamine amidation	aldehydes	• monosaccharide is attached to PS through C2 amidation
				• more reducing ends (C1 of the monosaccharide) introduced to PS
				• PS must have carboxyl groups

**Table 2:** Experimental Parameters, Green Aspects, Yields, And Maximum Oxidation Values of Various Approaches

reaction methods	experimental parameters	green aspects	yields (%)	maximum DS value or degree of oxidation
periodate oxidation	periodate ions (IO_4_^–^), water, rt, dark, min to h reaction times	aqueous, but high cost, low atom economy	>80%	91.5% oxidation^14^
bleach oxidation	bleach (NaOCl), water, acetic acid, rt, min to h reaction times	aqueous, relatively benign reagent, low cost	>80%	91.3% oxidation degree^15^
esterification with 4-formylbenzoates	4-formylbenzoic acid, dimethylformamide (DMF), dicyclohexylcarbodiimide (DCC), dimethylaminopyridine (DMAP), rt, N_2_, 24 h	expensive reagents, nonaqueous	not specified	not specified
acetoacetylation	diketene, TBAA, or THD, organic solvents (e.g., DMAc/LiCl) or ionic liquids; hours-long reaction	nonaqueous, but no coproducts with diketene; diketene-reactive, toxic	>80%	DS 2.91^16^
esterification with levulinate groups	levulinic acid, activation agent [DCC, TosCl, CDI or trifluoroacetic anhydride (TFAA)], organic solvents like DMAc, 80 °C, 24 h	not aqueous, reactive/toxic reagents	>80%	DS 2.42^17^
glucosamine amidation	glucosamine (GlcN), 4-(4,6-dimethoxy-1,3,5-triazin-2-yl)-4-methylmorpholinium chloride (DMTMM), water, rt, 24 h	aqueous, but DMTMM expensive	60%	DS 0.17* (alginate-GlcN)^16^

### 2.1 Oxidation

Oxidation strategies employ oxidizing agents (e.g., periodate ions or bleach) to convert polysaccharide hydroxy groups to aldehyde or ketone moieties. Two important but distinct methods have been developed that fall into this category: periodate oxidation and bleach oxidation. We discuss these two methods separately.

#### 2.1.1 Periodate Oxidation

**Scheme 1:**
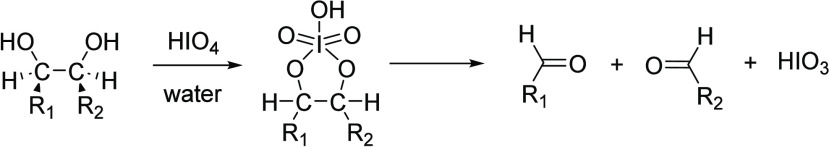
Mechanism of Periodate Oxidation of Diols

Periodate oxidation is a classical, versatile approach for rapid and efficient synthesis of polysaccharide aldehydes. For the chemistry to work, the polysaccharide must possess a vicinal diol entity, which is oxidatively cleaved by periodate to form an aldehyde moiety at each of the former vicinal hydroxy groups in the process of breaking the ring of that monosaccharide. This reaction was first discovered by Malaprade et al. in 1928 and has been widely used in carbohydrate chemistry since then because of many favorable factors,^17,18^ prominent among which is the high degree of regio- and chemoselectivity. Periodate oxidation can be restricted to vicinal 2,3-diols of polysaccharides under certain conditions.^19^ Periodate oxidation is widely used in polysaccharide chemistry because many important polysaccharides (e.g., amylose, cellulose, dextran) do contain vicinal diols and because the modification is one step and simple to carry out. Aqueous conditions are best for periodate oxidation, which suits polysaccharides well with their high water affinity (and in some cases, solubility). An accepted mechanism for periodate oxidation is shown in Scheme 1 in which diols form a cyclic intermediate with periodate ions, which decomposes to HIO_3_ and dialdehydes.^20^

**Scheme 2:**
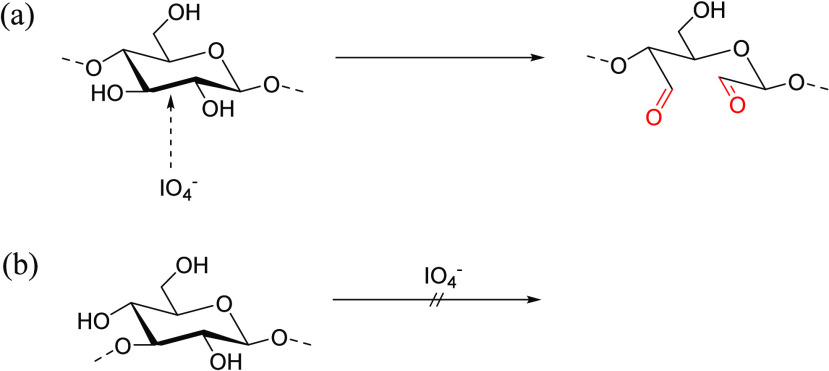
General Principles of Periodate Oxidation of Polysaccharides: (a) (1 → 4)-Linked Residues Where Cleavage Occurs Between C2 and C3 and (b) (1 → 3)-Linked Residues, Which Are Resistant to Periodate Oxidation Adapted with permission from ref (19). Copyright 2010 Elsevier.

For periodate oxidation to occur, it is required that the vicinal hydroxyls are oriented in such a way as to enable formation of the cyclic intermediate; either equatorial–equatorial or axial–equatorial orientation. Thus, periodate oxidation cannot take place if the vicinal −OH groups are in opposing, diaxial orientation to one another because they then cannot geometrically accommodate formation of the required cyclic intermediate. In addition, some important polysaccharides do not contain vicinal diols; for example, curdlan cannot be oxidized by periodate to a dialdehyde because its β-1,3 linkages mean that it contains no vicinal alcohols. General principles of periodate oxidation of polysaccharides using a →4)-β-D-Glcp-(1→ monosaccharide example (and illustrating the curdlan issue) are shown in Scheme 2.

**Scheme 3:**
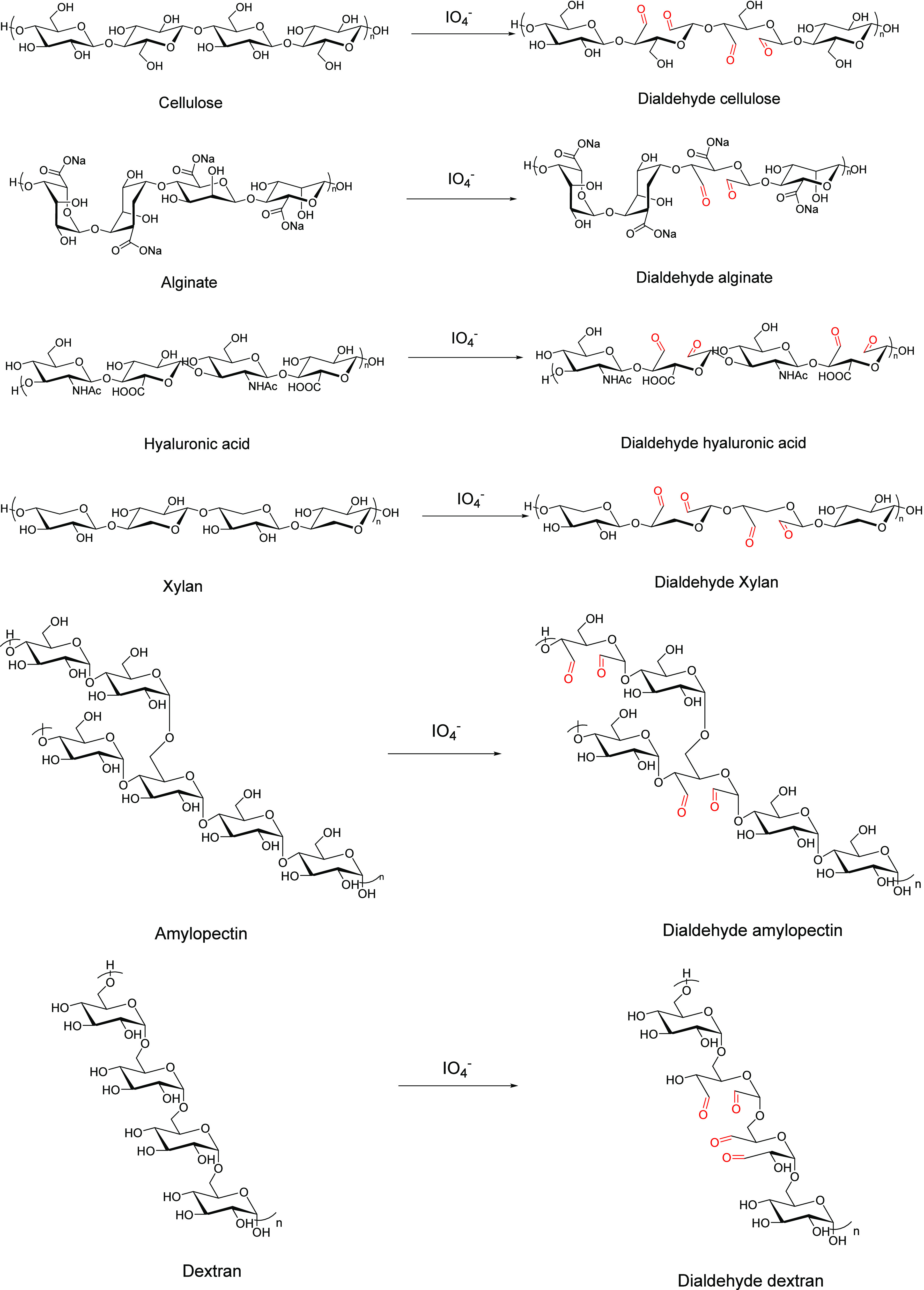
Illustration of Periodate Oxidation of Different Types of Polysaccharides

Various polysaccharides have been modified by the periodate oxidation method, including cellulose,^21−24^ alginate,^25,26^ dextran,^27,28^ starch,^29,30^ xanthan,^31^ glycosaminoglycans,^32,33^ and hyaluronic acid;^34−36^ examples are shown in Scheme 3.

Although periodate oxidation is chemo- and regioselective, the resulting vicinal dialdehydes have some complicating reactivities. It has been reported that these dialdehyde moieties are highly susceptible to alkaline β-elimination.^37^ The aldehydes are reactive with water and/or alcohols and so may be converted to hydrates, hemiacetals, or acetals. These can all be converted back to aldehydes relatively easily, but they greatly complicate product analysis since it is far easier to quantify intact aldehydes (aldehyde carbonyl in ^13^C or FTIR spectra, aldehyde proton in ^1^H NMR spectra being highly distinct) than, for example, hydrated aldehydes whose chemical shifts are very similar to those of other polysaccharide hydroxyl groups. More seriously, hemiacetal formation of the generated aldehydes with remaining polysaccharide hydroxyls can lead to undesirable properties. For example, dialdehyde cellulose is only soluble in hot water (>80 °C).^38^ Indeed, it must be recognized that interchain and intrachain cross-linking may occur.^14^ That is to say, the alcohol involved in conversion of the periodate-generated aldehydes to hemiacetals or acetals may arise from a separate polysaccharide chain or from another area of the same chain and lead to cross-linked or cyclic structures.

**Scheme 4:**
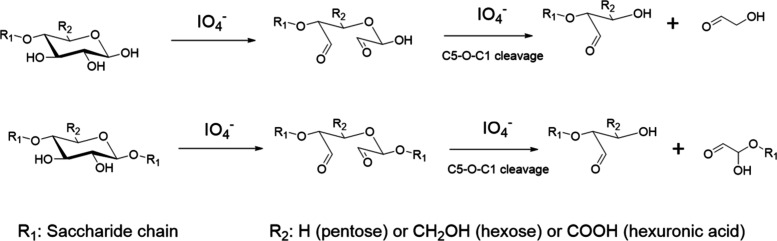
Degradation of Polysaccharide Chains Caused by Periodate Oxidation Reprinted or adapted with permission under a Creative Commons CC-BY license from ref (40). Copyright 2022 Elsevier.

Very importantly, since the monosaccharide rings of polysaccharides are inevitably opened by periodate oxidation, this will dramatically increase the polysaccharide chain flexibility. Under the conditions of the oxidation, some degradation will also occur, and the enhanced susceptibility of the oxidized product to further degradation reactions will also contribute to loss of mechanical properties, which will be undesirable for many applications.^19,39^ One degradation mechanism involves C5–O–C1 oxidative cleavage, while polysaccharide reducing ends of polysaccharides can also be oxidized (Scheme 4).^40^

#### 2.1.2 Bleach Oxidation

**Scheme 5:**
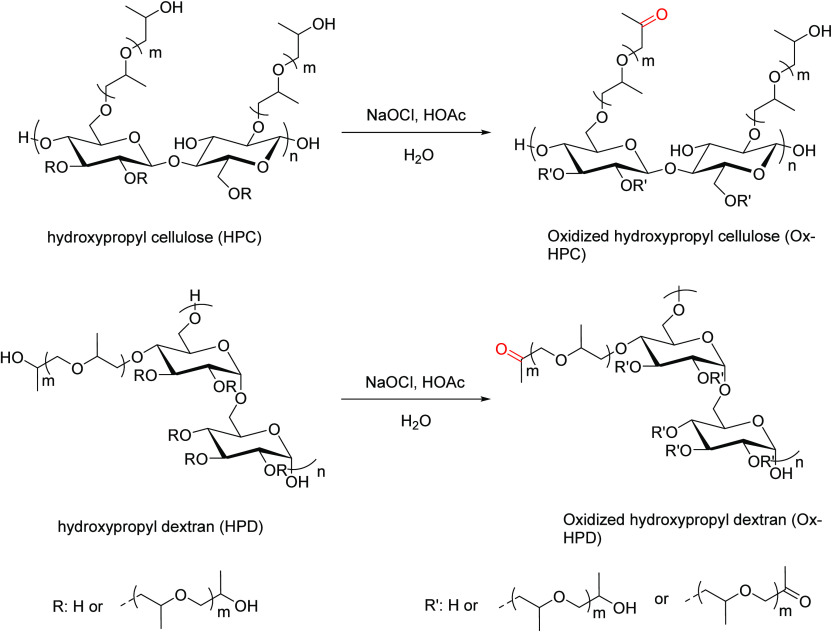
Bleach Oxidation of Hydroxypropyl Cellulose and Hydroxypropyl Dextran Adapted with permission from ref (42). Copyright 2020 American Chemical Society.

Oxidation of secondary alcohols to ketones was introduced for small molecule chemistry by Stevens et al.^41^ For small molecules, it is rapid, selective, and has the obvious attraction of employing an inexpensive reagent that is a common household cleaning agent. Application of small molecule chemistry to polysaccharides is often difficult, with issues of reactivity and selectivity. Thus, there was no report of application of bleach oxidation to ketones in polysaccharide chemistry for 40 years until Nichols et al. reported application of the chemistry to introduce ketone substituents to polysaccharides.^42^ Nichols et al. took advantage of the secondary alcohols at the termini of oligo(2-hydroxypropyl) substituents of hydroxypropyl (HP) ethers of polysaccharides, in particular, the commercial hydroxypropyl cellulose (HPC), as well as hydroxypropyl dextran (HPD), which was synthesized by the authors. Hydroxypropyl polysaccharides are readily prepared from the parent natural polysaccharide by an aqueous, alkaline reaction with propylene oxide. Reaction of the hydroxypropyl polysaccharide with aqueous bleach (sodium hypochlorite), ideally with pH adjustment using a small amount of acetic acid, oxidizes the terminal secondary alcohols of the oligo(2-hydroxypropyl) substituents to ketone groups. Mischnick in the Nichols publication showed by hydrolysis of the product to monosaccharides and GC/MS analysis that the oxidation was ∼90–95% selective for the oligo(hydroxypropyl) terminal secondary hydroxyls and nearly completely spared anhydroglucose ring hydroxyls. Bleach is alkaline, even after pH adjustment with acetic acid, so some loss of degree of polymerization occurs because of alkaline peeling. This can be moderated, and the DS (ketone) can be controlled by control of bleach stoichiometry (adding more bleach accelerates oxidation while not substantially accelerating DP loss). Bleach oxidations of hydroxypropyl polysaccharides are illustrated in Scheme 5.

Compared with periodate oxidation, bleach oxidation preserves the cyclic structure of monosaccharides, can be controlled to moderate DP loss, and does not introduce instability into the polysaccharide chain. It does require, unlike periodate oxidation, that the polysaccharide has been substituted with oligo(hydroxypropyl) moieties. Both oxidations can be readily controlled stoichiometrically. The ketones that result from bleach oxidation are less prone to undesired further reactions (e.g., cross-linking via acetal formation) but also are less reactive toward nucleophiles like amines than are the highly reactive aldehydes. Even so, the ketone substituents introduced by bleach oxidation have already been employed for further conversions, like reductive amination, to append bioactive small molecules and cross-linking with amine-containing polymers to readily prepare injectable hydrogels, including all-polysaccharide hydrogels.^15,43−45^ Overall, bleach oxidation of hydroxypropyl polysaccharides is simple and selective and introduces highly useful reactivity.

### 2.2 Nonoxidative Modification

Nonoxidative modification refers to the reaction of small molecules with polysaccharides to introduce aldehyde or ketone-containing substituents directly to the polysaccharide, typically by reactions in which the polysaccharide is the nucleophile. Three types have been reported, and illustrative examples will be described here: esterification with 4-formylbenzoates, acetoacetylation, and esterification with levulinate groups.

#### 2.2.1 4-Formylbenzoic Acid Esterification

**Scheme 6:**
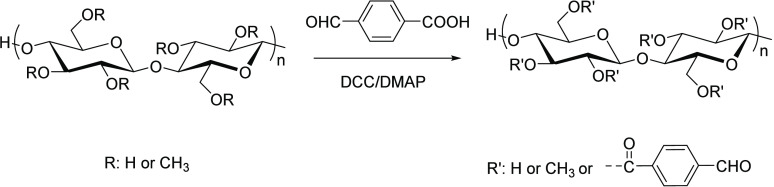
Esterification of Methyl Cellulose with 4-Formylbenzoic Acid Adapted with permission from ref (46). Copyright 2020 Royal Society of Chemistry.

4-Formylbenzoic acid is a byproduct of terephthalic acid synthesis by oxidation of *p*-xylene and is, thus, readily available and relatively inexpensive. Researchers have approached esterification of polysaccharides with 4-formylbenzoate by using the carboxylic acid, itself (rather than activated derivatives, such as the acid chloride or anhydride), in conjunction with a condensation reagent, such as dicyclohexyl carbodiimide (DCC), in the presence of dimethylaminopyridine (DMAP) (Scheme 6). Wang et al.^46^ modified methyl cellulose by acylation with 4-formylbenzoic acid and further fabricated self-healing Schiff base hydrogels by reaction with PEG-grafted chitosan. This approach is not necessarily regioselective and does require DCC or a similar condensing agent to work.

So far, only one example using 4-formylbenzoic acid esterification to synthesize polysaccharide aldehydes has been published, but the method should be equally useful for other types of polysaccharides. In addition, the general approach is attractive: that of using a difunctional small molecule reagent in which one end can be appended to the polysaccharide by simple, well-understood reactions (e.g., formation of ether, ester, or carbamate bonds), and the other end bearing a ketone or aldehyde group. Indeed, it could be even more useful if the aldehyde were protected in such a way as to be readily deprotected when needed, thus potentially minimizing interference by undesired cross-linking. It is likely that this strategy will be more widely explored in the near future.

#### 2.2.2 Acetoacetylation

**Scheme 7:**
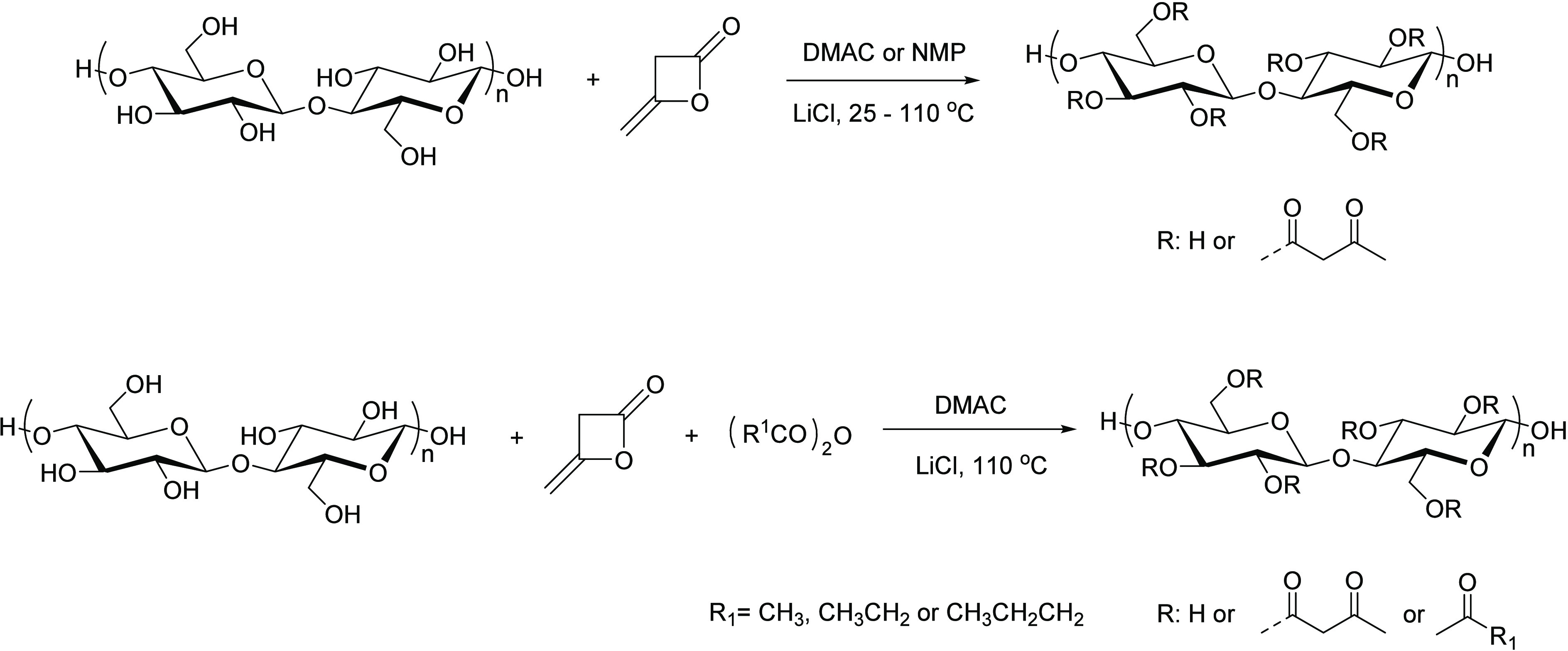
Synthesis of Cellulose Acetoacetates and Cellulose Acetoacetate Alkanoates Adapted with permission from ref (49). Copyright 1995 American Chemical Society.

Acetoacetylation has been well-studied for small molecules by typically employing reaction of diketene (CH_2_=C=O) with nucleophiles, like alcohols or amines, to form acetoacetate (AcAc) esters or amides.^47^ Diketene is produced by the thermal dehydration of acetic acid. Its reactions with polysaccharides have been studied previously to a limited extent. Staudinger and co-workers^48^ reported heterogeneous reaction of amorphous, regenerated cellulose with diketene in acetic acid using sodium acetate as catalyst. Elemental analysis results confirmed that they achieved a DS(AcAc) of 3. Edgar et al.^49^ reported homogeneous reaction of microcrystalline cellulose with diketene in *N*,*N*-dimethylacetamide (DMAC)/LiCl or n-methyl-2-pyrrolidinone (NMP)/LiCl solution. Reaction with alkanoyl chlorides or alkanoic anhydrides could also be accomplished in the same solution to afford near-quantitative conversion of both diketene and the other acylation reagent (Scheme 7). Thus, cellulose acetoacetates and cellulose acetoacetate alkanoates with a wide variety of DS values were obtained in this way; the methodology affords access to the complete range of DS values and to a very broad range of mixed cellulose acetoacetate alkanoate esters. It would be expected that this chemistry would also apply to a broad range of other polysaccharides.

**Scheme 8:**
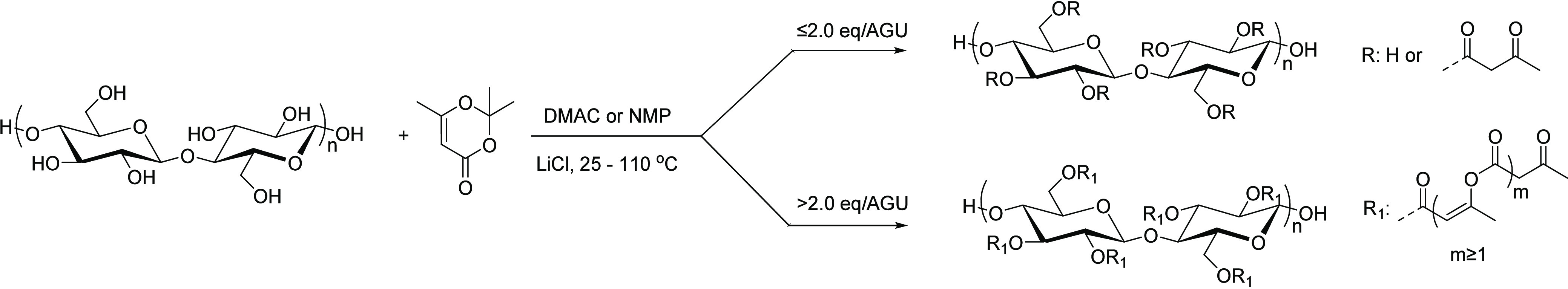
Acetoacetylation of Cellulose with 2,2,6-Trimethyl-4*H*-1,3-dioxin-4-one (THD) Adapted with permission from ref (52). Copyright 2018 Springer.

Diketene is an excellent reagent for acetoacetylation because it is inexpensive, quite reactive toward nucleophiles, and is a liquid that can be readily handled by using appropriate care. However, diketene is also a lachrymator, is relatively volatile, and is highly reactive, including with water. As a result, shipment of diketene is prohibited in some countries, including the United States. Because of the challenging features of diketene, derivatives have been developed that are not lachrymators and are not overly reactive at room temperature; they are designed to decompose to generate diketene upon heating. Useful derivatives for acetoacetylation include *tert*-butyl acetoacetate (TBAA)^50^ and 2,2,6-trimethyl-4*H*-1,3-dioxin-4-one (THD).^51^ Würfel et al. reported homogeneous, catalysis-free synthesis of cellulose acetoacetates using THD to afford cellulose acetoacetates with various DS(AcAc) values when the molar ratio of THD was less than 2 equiv per anhydroglucose unit (AGU).^52^ When that molar ratio was >2, the reaction led to enol ester formation and, hence, to a degree of molar substitution (MS) that could exceed 3 (Scheme 8). Reaction with TBAA, however, is initiated thermally above approximately 100 °C where TBAA decomposes to *t*-butyl alcohol and diketene, which can react with polysaccharide alcohols.^53^

Acetoacetylation is an efficient and versatile approach for appending ketone functionality to polysaccharides. The approach is given special characteristics, some useful and some not, by the particular reactivity of the acetoacetate ketone group, which is β to an ester group. The acetoacetate group can react with amines to form enamines; enamine formation is dynamic and reversible in the presence of water. What’s more, the two electron-withdrawing groups (ketone and ester) make the α-carbon protons (α to both ketone and ester groups) more acidic, such that they can be easily deprotonated by a base. The resulting anion is a nucleophile that can react with acrylates,^53,54^ isocyanates^55^ and diazonium salts^56^ to afford different functionalities that may be useful in various applications. Other interesting reactions involving the acetoacetate group have been demonstrated, including the Biginelli^57^ and Hantzsch^58^ reactions.

It is important to note that the resulting polysaccharide acetoacetate esters have limited thermal stability because of the potential for thermal reversion to acetyl ketene.^50^ This thermal reversion may be undesirable in some uses (e.g., thermoplastics) but desirable in others (perhaps for biodegradable materials, for example). While publications to date have been focused on cellulose acetoacetylation, clearly any polysaccharide that has hydroxy groups (that is to say, any natural polysaccharide) or amino groups could be acetoacetylated using one or more of these methods.

#### 2.2.3 Levulinate Esterification

**Scheme 9:**
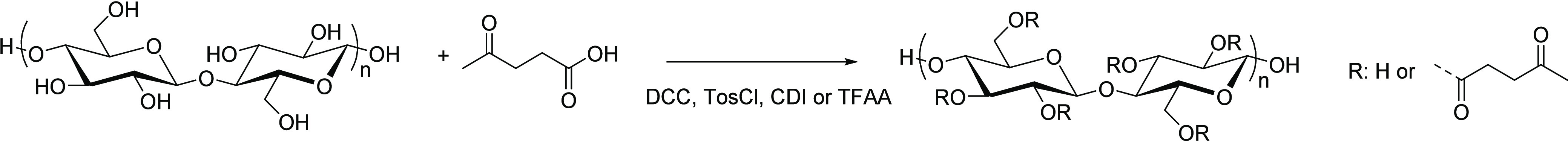
Levulinate Esterification of Cellulose Using Mild Activation Methods Adapted with permission from ref (62). Copyright 2015 Springer.

Levulinic acid is, itself, a sustainable material available by acid-catalyzed hydrolysis of cellulose. Levulinates are difunctional (Scheme 9) by containing both a ketone and a carboxylic acid.^59,60^ Levulinate esters are commonly used as protecting groups in carbohydrate chemistry because they are acid-stable and can be easily removed by reaction with hydrazine. This selectivity arises because difunctional hydrazine can react simultaneously and favorably (five-membered ring intermediate) with both the levulinate ketone and ester carbonyls.^61^ As noted earlier, transposition of small molecule reactions to polysaccharides can be challenging, as exemplified by levulinates; there are few examples of synthesizing polysaccharide ketones by levulinate esterification. In fact, the only example was reported by Zheng et al.,^62^ who explored synthesis of cellulose levulinates in detail. Having identified a number of approaches that work for small molecule carbohydrates but not for polysaccharides, they identified methods to synthesize cellulose levulinates by the mild activation of levulinic acid (Scheme 9).

Polysaccharide levulinates are usually more thermally stable than polysaccharide acetoacetate esters. The reactivity of the ketone group is more similar to that of a typical ketone than to that of the acetoacetate ketone since the ketone and ester are not β to one another in levulinate moieties. The levulinate ester carbonyl carbon is three atoms from the ketone and is, therefore, less influential upon it. The main issue impeding the broader use of this approach is that levulinate esterification is synthetically somewhat challenging and can be plagued with side reactions due to the relatively poor reactivity of polysaccharide alcohols.

### 2.3 Multireducing End Modification

**Scheme 10:**
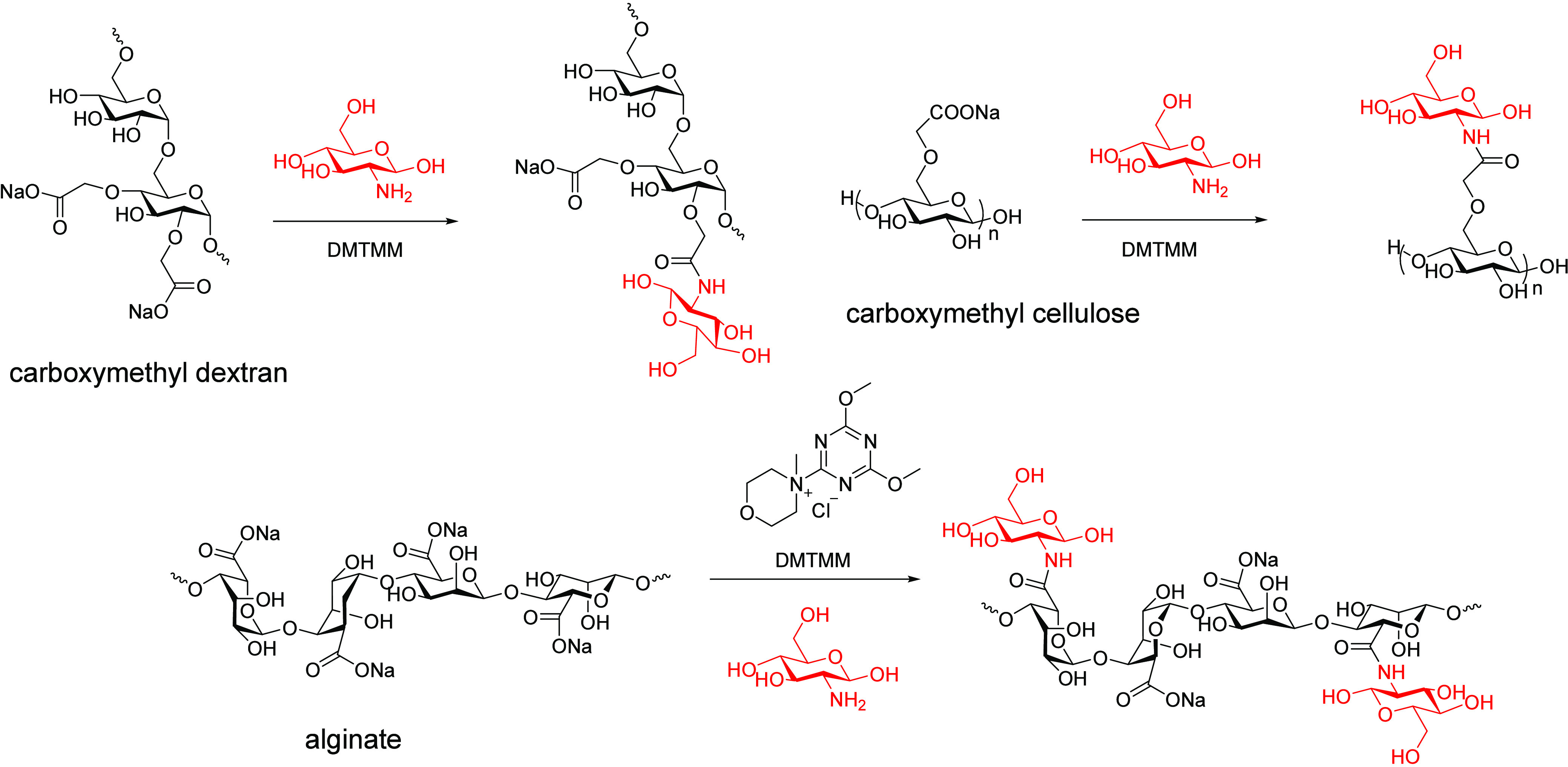
Reducing End Modification of Carboxymethyl Dextran, Carboxymethyl Cellulose, and Alginate to MREPs Adapted with permission from ref (16). Copyright 2023 American Chemical Society.

Since every natural aldose-based polysaccharide has only one reducing end and, thus, only one carbon that is in equilibrium between aldehyde and hemiacetal, the ability to use that aldehyde for appending substituents is very limited and does not provide the multifunctionality necessary for preparation of useful entities, like aldehyde-linked networks (hydrogels) or triblock copolymers. For this reason, investigators have recently introduced the concept of multireducing end polysaccharides (MREP). MREPs have been prepared by the attachment of a monosaccharide to the polysaccharide through linkages from positions on the added monosaccharide other than C1. In this way, each C1 aldehyde (reducing end) that is appended remains free for reactions, like imine formation or reductive amination, thereby affording a multialdehyde functional polysaccharide derivative. MREPs were first reported by Zhai et al.,^16^ who utilized amide formation between the carboxylic acids of poly(uronic acids) (e.g., alginate) or carboxymethyl-substituted polysaccharides (e.g., carboxymethyl cellulose) with the amine moieties of glucosamine or galactosamine to synthesize MREPs (Scheme 10). NMR spectroscopy, fluorimetry, and the silver mirror reaction all confirmed that a significant DS of monosaccharides, each with an added reducing end, could be appended to the polysaccharides. The desired added aldehyde reactivity needed to be confirmed since each added aldehyde is in equilibrium with its cyclic, hemiacetal form; would they react like a simple aldehyde (e.g., like acetaldehyde)? Zhai and co-workers demonstrated that their MREPs could indeed form hydrogels at room temperature with amine-containing polymers, like polyethylenimine (PEI), in part because of the formation of imine cross-links.

Compared with periodate oxidation, multireducing end modification can also introduce many aldehyde groups to polysaccharides, thereby controlling stoichiometry by controlling the amino monosaccharide/polysaccharide ratio, while keeping the monosaccharides of the polysaccharide intact, largely preserving DP, and avoiding the introduction of instability. However, the DS of multireducing end modification obtained to date has been relatively low. New chemistry is needed to improve the DS (aldehyde) and, thus, the reactivity and utility potential of the multireducing end modification approach.

## 3 Reactivity

In this section, we briefly discuss the reactivity of polysaccharide aldehydes and ketones and introduce applications of these materials. Since there have been a number of reviews regarding polysaccharide applications,^3,6,44,63−69^ we will focus on their reactivity. Reactions of aldehyde- and ketone-substituted polysaccharides reported in the literature are summarized in Table 3.

**Table 3:** Reactions and Applications of Polysaccharide Aldehydes and Ketones

reaction	functional groups involved	related applications
Schiff base formation	aldehyde or ketone; amine	1. injectable and self-healing PS hydrogels
		2. drug delivery vehicles
		3. sensor–adsorbent for mercury ions
reductive amination	aldehyde or ketone; amine	1. PS–protein conjugate
		2. renewable thermoplastics.
enamine formation	acetoacetate; amine	1. injectable, responsive, and self-healing PS hydrogels
		2. surface modification of sponges or fabrics for oil/water separation or antibacterial wound dressing
horseradish peroxidase (HRP)-mediated polymerization	acetoacetate; vinyl monomers	graft polymerization on cellulose
Biginelli reaction	acetoacetate; aldehyde; urea	cellulose PEGylation
Hantzsch reaction	aldehyde; acetoacetate; ammonium acetate or ammonia	cellulose film for UV-blocking

### 3.1 Schiff Base Formation

**Scheme 11:**
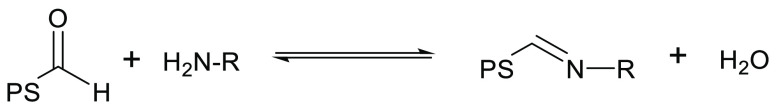
General Synthetic Scheme for Schiff Base Formation: Aldehyde Group from Polysaccharides (PS) Reacts with Small Molecule or Polymer-Bearing Amine Group to Afford an Imine Bond

Schiff base formation is perhaps the most widely used and studied reaction for polysaccharide aldehydes and ketones. It refers to the reversible condensation of an aldehyde or ketone with a primary amine to form an imine bond, which generates a molecule of water as byproduct (Scheme 11).

One useful, widely studied application of Schiff base formation is the fabrication of injectable, self-healing polysaccharide hydrogels. Reversible imine bonds (especially in the water-rich hydrogel environment) provide the ability to self-repair, which, in turn, provides injectability. This can not only facilitate hydrogel extrusion from a syringe and solidification at the targeted position but can also provide for timely self-repair of structural defects.^70,71^ Several recent reviews cover the details of synthesis and application of injectable, self-healing polysaccharide hydrogels.^63,64^

**Scheme 12:**
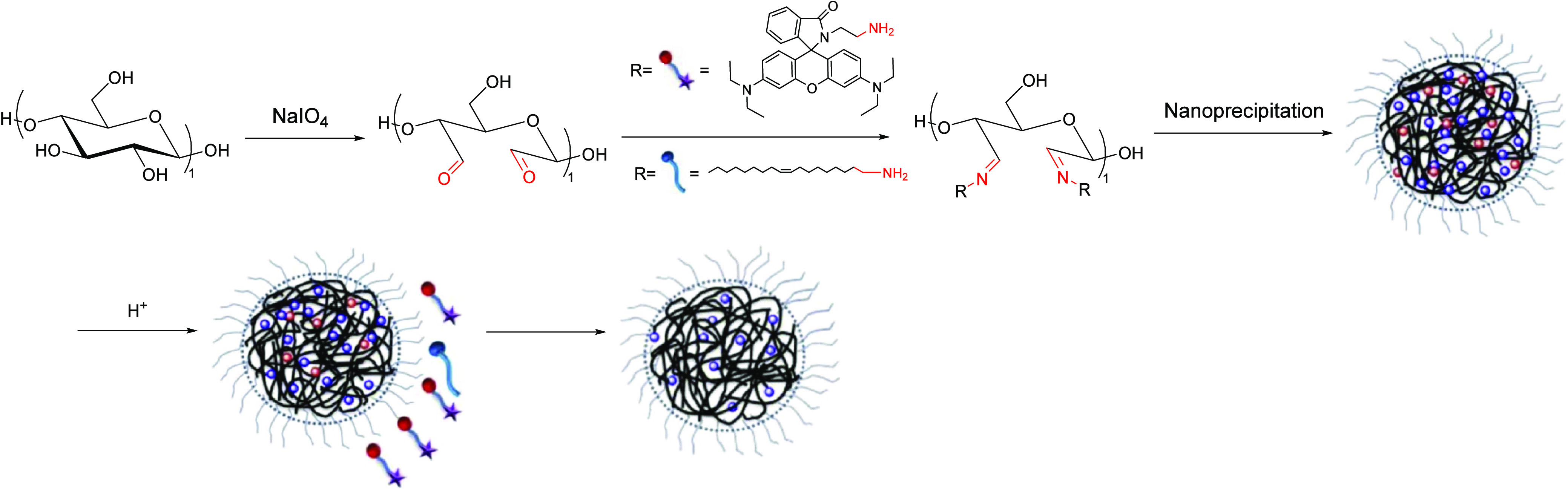
Schematic Representation for Formation of pH-Responsive Nanoparticles Adapted with permission from ref (76). Copyright 2019 Elsevier.

Schiff bases formed from aldehyde- or ketone-substituted polysaccharides have also been used to fabricate drug delivery vehicles. We will provide only a couple of illustrative examples since there are several comprehensive reviews of this field.^72−75^ Peng et al. reported a pH-responsive nanoparticle from cellulose aldehydes via Schiff base formation for controlled release.^76^ The process began with periodate oxidation of cellulose to generate ring-opened dialdehydes, which were then conjugated with oleylamine and aminoethyl Rhodamine via imine bonds. Nanoparticles were formed by precipitation into water (Scheme 12). Because of the pH-responsive imine bonds, which are more labile under acidic conditions (pH < 5), the nanoparticles evinced faster release at pH 4 compared with pH 5 or pH 7.4.

**Scheme 13:**
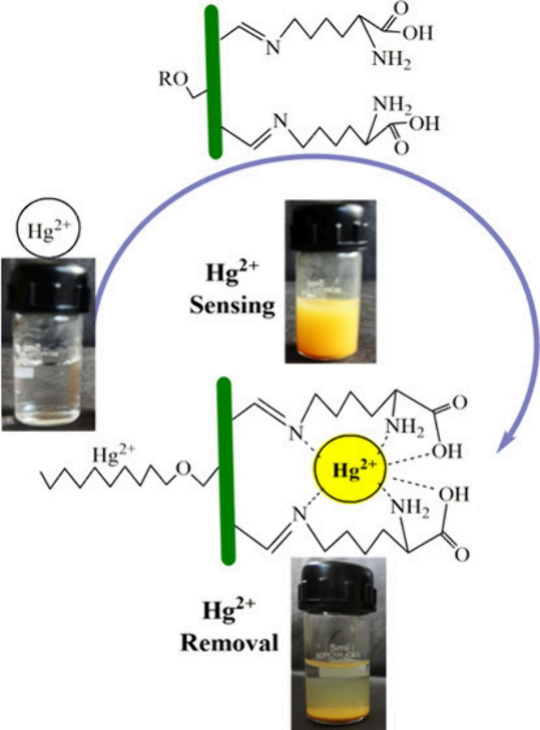
Schematic Representation of Sensing and Removal of Mercury Ions Using Cellulose–Lysine Schiff Base Reproduced with permission from ref (77). Copyright 2014 American Chemical Society.

Researchers have exploited Schiff base chemistry to append substituents that enable the design of polymers with dual-sensor and absorbent capability for mercury ions, thereby demonstrating the power of functionalization with pendant aldehyde moieties. Kumari et al. reported a novel cellulose–lysine Schiff base for mercury ion sensing and removal.^77^ Cellulose was first extracted from powdered pine needles and reacted with aliphatic bromides to form cellulose ethers, and then, those cellulose ethers were oxidized by periodate ions to generate ring-opened dialdehyde moieties on the monosaccharides that still had unsubstituted vicinal 2,3-diols. Finally, lysine was conjugated with those aldehyde moieties to form lysine-substituted cellulose derivatives via Schiff base linkages. Mercury ions formed colored complexes with pairs of proximate Schiff base-linked lysines because of the reaction mechanism in which two nearby aldehydes are generated synchronously. The mercury ions could be removed from the resulting complex by treatment with dilute HCl (Scheme 13).

### 3.2 Reductive Amination

**Scheme 14:**
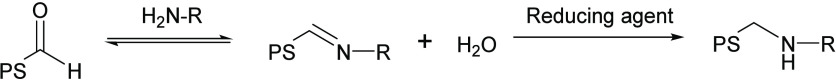
General Synthetic Scheme for Reductive Amination: Aldehyde Group from Polysaccharides (PS) Reacts with Small Molecule or Polymeric Primary Amines to Afford Imine Bonds, Which Are Then Reduced to Secondary Amine

Reductive amination involves initial Schiff base formation; subsequently, the imine bond formed by the primary amine reaction with aldehyde or ketone is reduced to a secondary amine. The product secondary amines are more stable than the imine intermediates because they are far more resistant to hydrolysis. Reduction can be of the isolated imine, or the imine formation and reduction can be conducted in a one-pot operation (more common since it avoids isolation of the hydrolytically labile imine). One-pot reductive amination demands a selective reducing agent that can effectively discriminate between reduction of the starting aldehyde and the intermediate imine. Reducing agents commonly employed for this purpose include borohydrides, like sodium borohydride (NaBH_4_), sodium cyanoborohydride (NaBH_3_CN), and sodium triacetoxyborohydride [NaBH(OAc)_3_],^78^ where the latter two are typically more chemoselective. The general reductive amination reaction scheme is shown in Scheme 14.

**Scheme 15:**
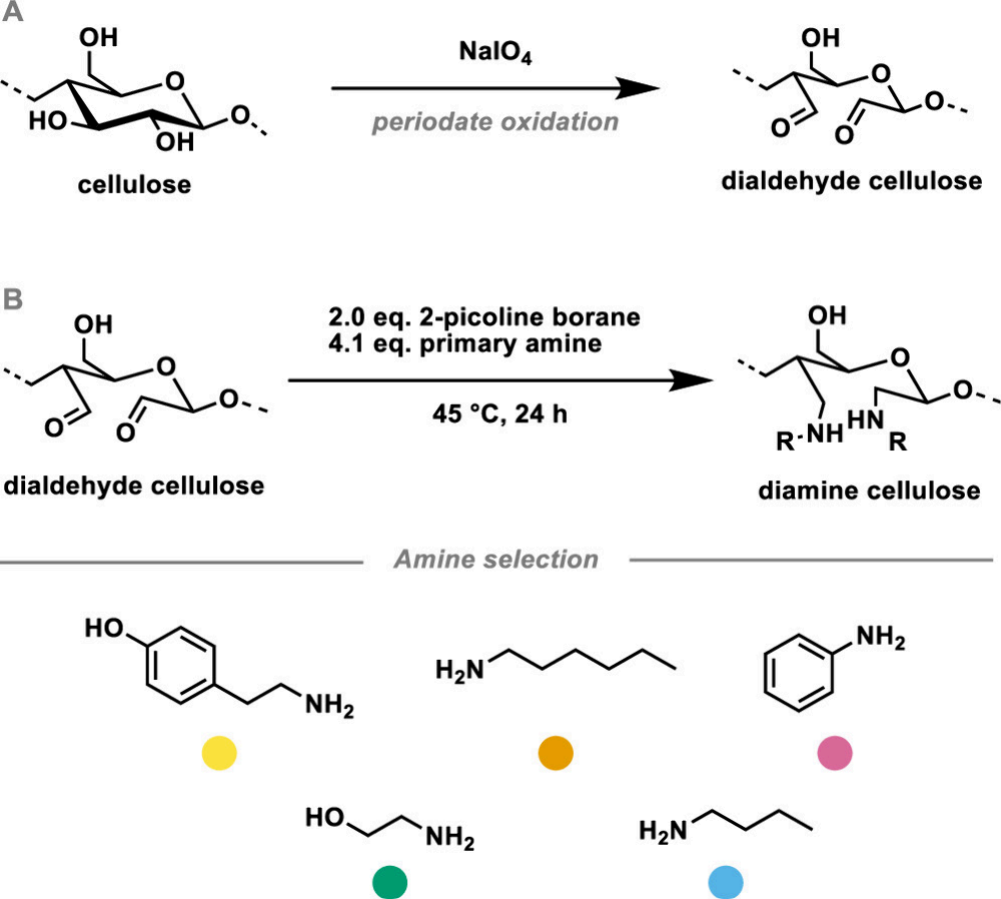
Synthesis Procedure for Cellulose Diamine Whereby Cellulose Was First Oxidized by Periodate to Generate Cellulose Dialdehyde and Then Reductively Aminated with Five Different Primary Amines Reproduced with permission from ref (80). Copyright 2023 American Chemical Society.

Reductive amination has been used widely for the synthesis of prodrugs or for making polysaccharide–protein conjugates. Detailed descriptions can be found in the reviews of refs (67 and 79). The design of renewable thermoplastics is another application of the reductive amination of polysaccharide aldehydes or ketones. Simon et al. reported using periodate-oxidized cellulose dialdehyde to react with amine small molecules to make cellulosic diamines through reductive amination.^80^ Five primary amines, including both aliphatic and aromatic, were introduced to the cellulose backbone to tune the glass-transition temperature (*T*_g_). Although these transformations were accompanied by degradative side reactions, like β-elimination and incomplete conversion during reductive amination, dianiline cellulose showed the highest conversion, the best thermal properties, and a peculiar symmetrical molecular weight distribution (Scheme 15).

### 3.3 Enamine Formation

**Scheme 16:**
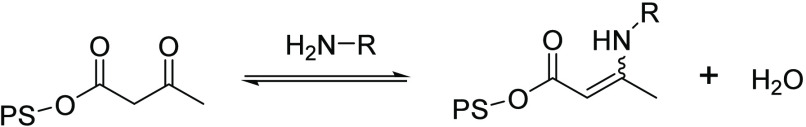
General Scheme for Enamine Formation: Acetoacetate Group from Polysaccharide (PS) Reacts with Small Molecule or Polymeric Secondary Amines to Afford Enamine Bonds

Broadly, enamine formation most often is the result of a reaction between an aldehyde or ketone and a secondary amine. However, in the context of this review, we restrict our discussion of enamine formation to reaction between polysaccharide acetoacetates and primary amines (Scheme 16).

**Scheme 17:**
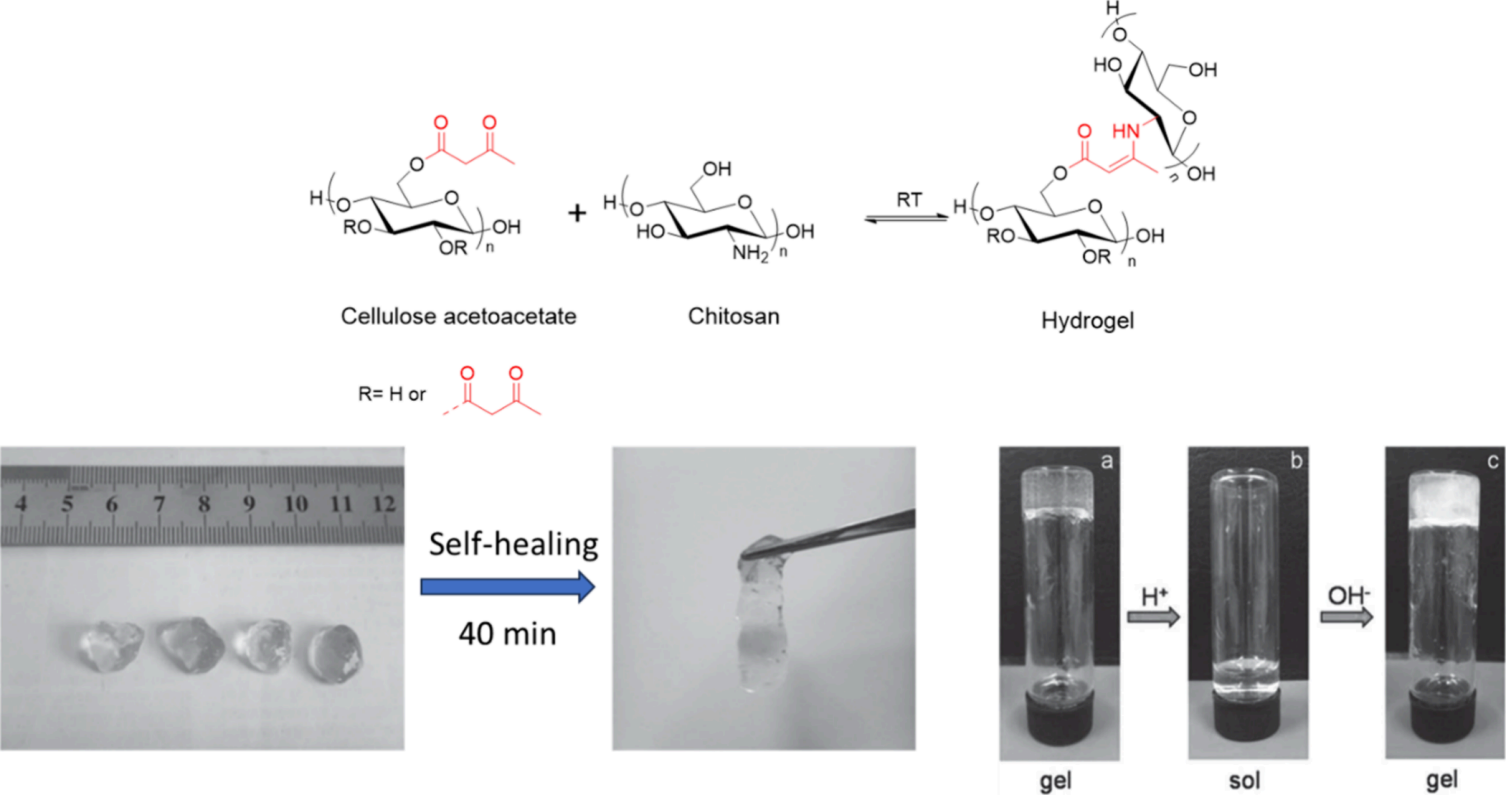
Synthetic Scheme for Enamine-Based Polysaccharide Hydrogel (Above) and Self-Healing and pH-Responsive Properties of the Hydrogel (Below) Adapted with permission from ref (81). Copyright 2016 Wiley.

Liu et al. reported fabrication of a self-healing polysaccharide hydrogel on the basis of dynamic covalent enamine bonds.^81^ The polysaccharide hydrogel was obtained by mixing aqueous solutions of cellulose acetoacetate (CAA) and chitosan at room temperature (Scheme 17). The resulting hydrogel exhibited self-healing and pH-responsive properties because of the dynamic and reversible enamine bonds, which decompose more rapidly in acidic environments.

**Scheme 18:**
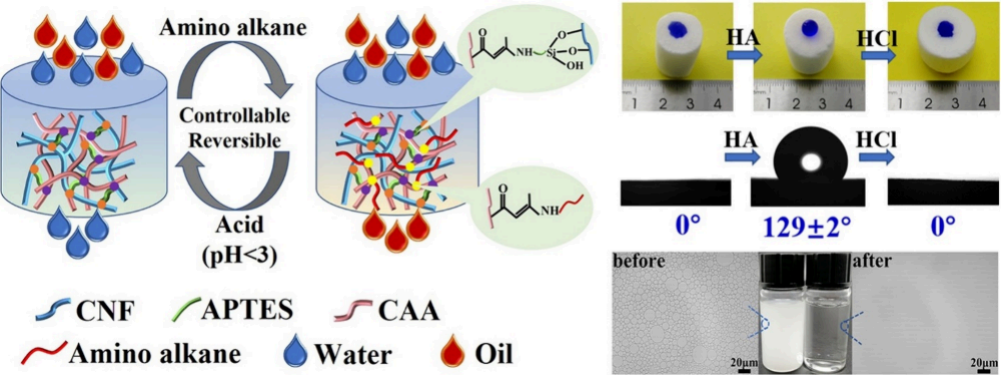
General Scheme for Controllable Water/Oil Separation of Functionalized CAA Sponge via Enamine Formation Reproduced with permission from ref (82). Copyright 2020 Elsevier.

Formation of enamines on the basis of cellulose acetoacetate can also be used for surface modification of sponges or fabrics for oil/water separation or antibacterial wound dressing. Li et al. reported fabrication of a functional porous cellulose acetoacetate (CAA) sponge by cross-linking cellulose acetoacetate with (3-aminopropyl)triethoxysilane (APTES) in the presence of cellulose nanofibers (CNF).^82^ The CAA sponge could be easily modified by alkylamines [e.g., hexylamine (HA)] of varying carbon chain length via dynamic covalent enamine bonds. Hydrophilicity of CAA sponges could be tuned readily from very hydrophilic to highly hydrophobic under suitable pH conditions because of the dynamic and reversible enamine bonds. High and selective oil absorption capacity (40–80 g/g) and satisfying desorption ability of 80% could be achieved by alkyl-functioned CAA sponges. The investigators reported that the CAA sponges could also efficiently separate oil–water mixtures and emulsions (>99% of water or oil could be separated from each other) in a controllable manner (Scheme 18).

**Scheme 19:**
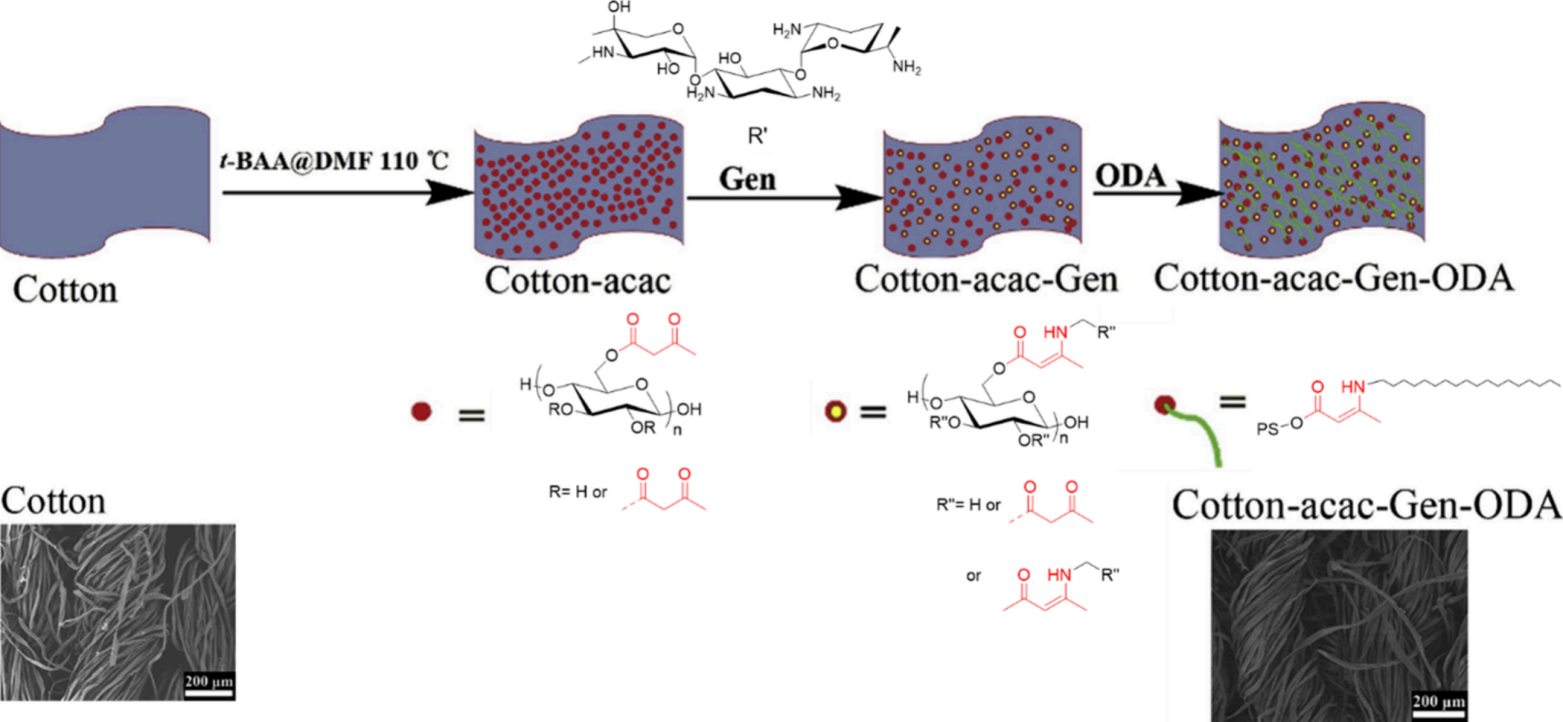
General Scheme Illustrating Preparation of Antibacterial and Hydrophobic Cotton Fabric Adapted with permission from ref (83). Copyright 2019 Elsevier.

A similar strategy was employed by Rong et al., who modified cotton fabric with acetoacetyl groups and anchored antibacterial gentamicin (Gen) and hydrophobic octadecyl amine (ODA) by enamine bonds in order to impart dual functions (Scheme 19).^83^

### 3.4 Horseradish Peroxidase (HRP)-Mediated Polymerization

**Scheme 20:**
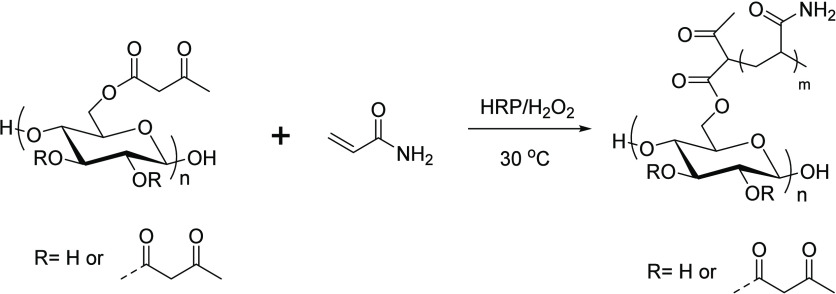
Synthetic Illustration for Graft Polymerization from Cellulose Acetoacetate Adapted with permission from ref (53). Copyright 2021 Springer.

Horseradish peroxidase (HRP) can catalyze the reaction between β-keto carbonyl compounds and hydrogen peroxide (H_2_O_2_) to abstract a labile hydrogen atom from between the carbonyls to form a carbon-centered radical, thereby enabling initiation of radical polymerization of vinyl monomers.^84−86^ This method was successfully applied to cellulose acetoacetate for efficient graft polymerization by Wang et al.^53^ Monomers of various reactivities, polarities, and functionality were used for graft polymerization on CAA, including acrylamide, 2-hydroxyethyl methacrylate (HEMA), methyl methacrylate (MMA), and sulfobetaine methacrylate (SBMA) (Scheme 20).

### 3.5 Biginelli Reaction

**Scheme 21:**
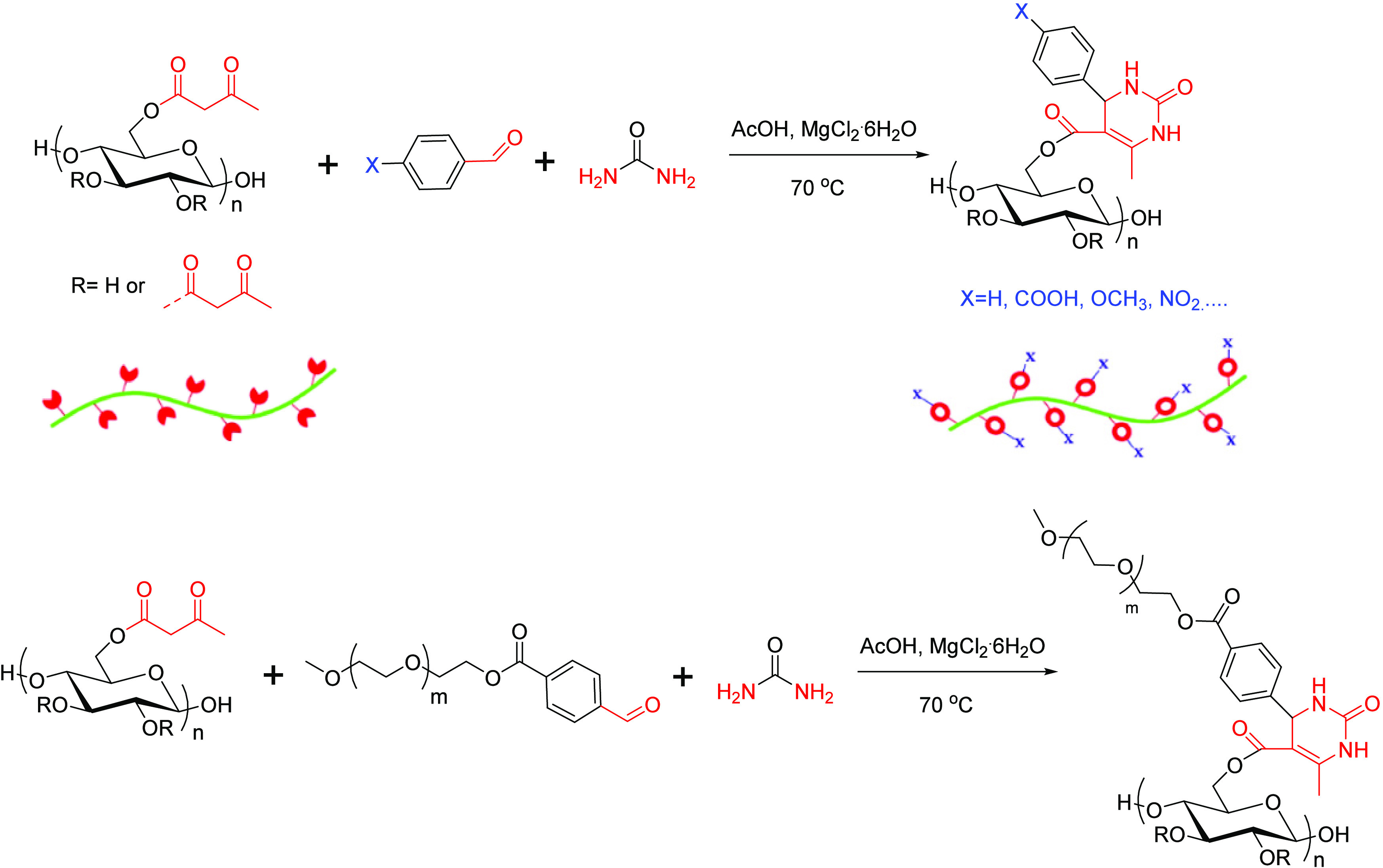
Synthesis Procedure for Cellulose-Based Derivatives Using Biginelli Reaction Adapted with permission from ref (57). Copyright 2019 Elsevier.

The Biginelli reaction is a multicomponent, one-pot condensation of acetoacetate, aldehyde, and urea or thiourea to form a cyclic structure. It is a versatile, efficient reaction for generating complex structures quickly that is able to employ a wide scope of substrates.^87^ Rong et al. reported using the Biginelli reaction for generating cellulose derivatives.^57^ A library of cellulose-based materials with different functional groups was successfully synthesized by applying the Biginelli reaction to cellulose acetoacetate (Scheme 21). A good example of the properties that can be imparted to a polysaccharide in this way is shown in Scheme 15B, where the Biginelli reaction provides a convenient way to append poly(ethylene glycol) monomethyl ether (mPEG) substituents to the cellulose chain mildly and efficiently, thereby providing a simple way to enhance the water solubility of the products (while imparting other structural features and properties simultaneously).

### 3.6 Hantzsch Reaction

**Scheme 22:**
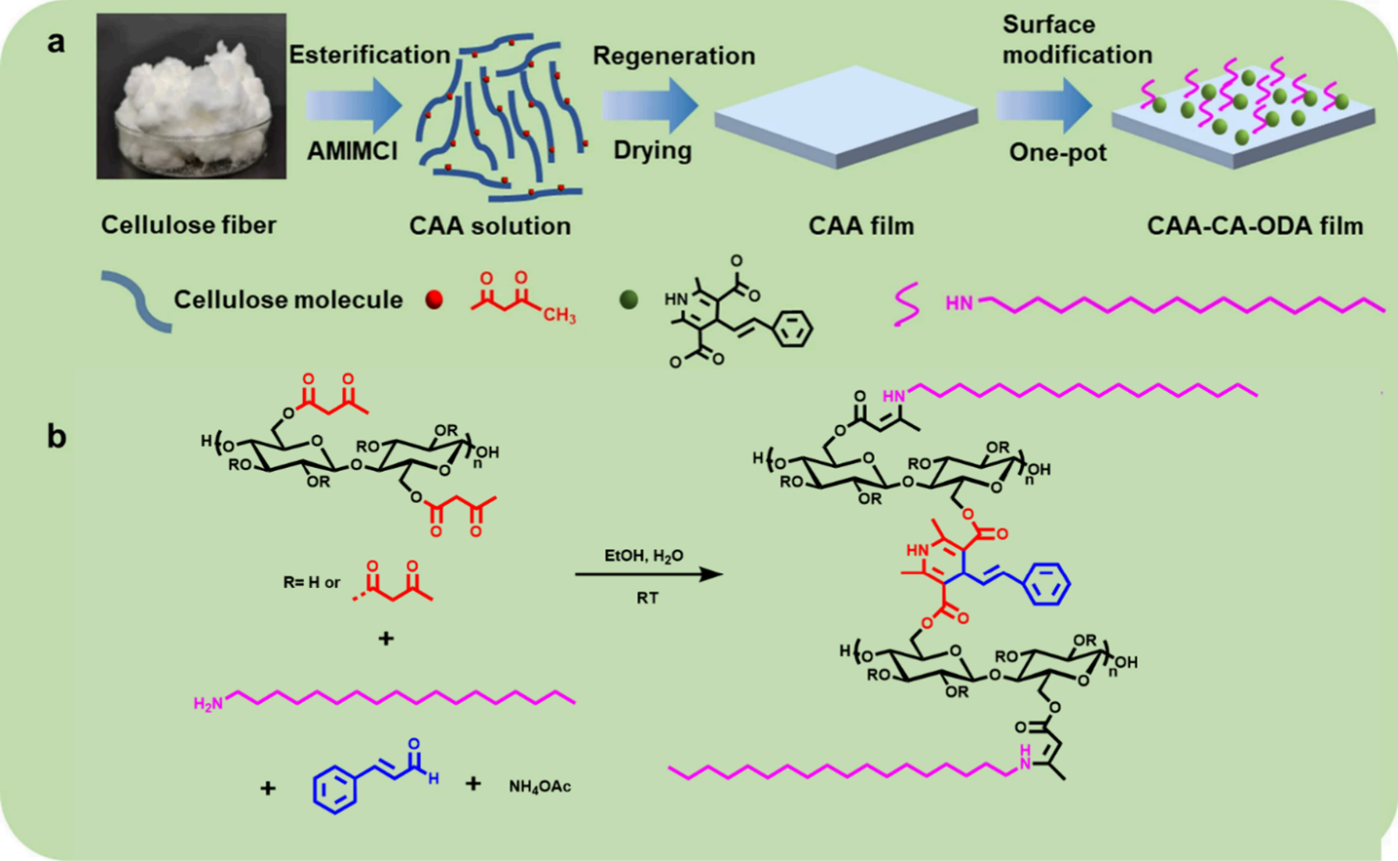
(a) Process for the Fabrication of Hydrophobic, UV-Blocking Cellulose Film and (b) Synthetic Procedure for Cellulose-Based Derivatives Using Hantzsch Reaction Adapted with permission from ref (58). Copyright 2022 Springer.

Like the Biginelli reaction, the Hantzsch reaction is a one-pot, multicomponent condensation reaction, in this case, involving acetoacetate, aldehyde, and ammonia. Qiu et al. applied the Hantzsch reaction to cellulose acetoacetate to fabricate fluorescent and hydrophobic cellulose-based films for full-band UV-blocking.^58^ The natural hydrophobic and UV-absorbing molecule cinnamyl aldehyde (CA) was used to build 1,4-dihydropyridine (DHP) fluorescent rings by the Hantzsch reaction to improve the UV-blocking performance of the cellulose film. In addition, hydrophobic long-chain octadecylamine (ODA) moieties were incorporated through enamine formation to enhance the film hydrophobicity (Scheme 22).

## 4 Conclusions and Outlook

Synthesizing polysaccharides possessing aldehyde or ketone groups is enabling many applications because of the rich and specific reactivity of those groups. Although a number of strategies have been described by previous researchers for preparation of polysaccharide aldehydes or ketones, as elucidated in this review, synthetic challenges remain as detailed below.

Periodate oxidation, as the most widely used method to prepare polysaccharides bearing aldehyde groups, does have important advantages, including efficiency; simplicity (one step, no protecting groups needed); and regio- and, to some degree, chemoselectivity. However, as noted above, periodate oxidation of a polysaccharide breaks down the cyclic structure of those monosaccharides that are oxidized (degrading desirable physical properties for most applications), reduces DP, and creates instability that can lead to further DP loss or other side reactions as the material is stored or used.^19^ Undesired side reactions of the formed free aldehyde groups can include alkaline β-elimination, as well as inevitable chemistry of the dialdehyde functionality, such as hydration or hemiacetal formation with hydroxy groups on the same chain or on other molecules (leading to cross-linking).^14,37^ In addition, certain important polysaccharides (e.g., 1,3-glucans) do not possess vicinal diols and so are not substrates for periodate oxidation.

Bleach oxidation of terminal secondary alcohols of oligo(hydroxypropyl) substituents of polysaccharides is attractive since no rings are broken, the method is highly chemo- and regioselective, aqueous NaOCl is cheap and readily available, methods exist to control DP loss, and such HP derivatives (including commercial cellulose HP ethers, such as HPC, hydroxypropyl methyl cellulose, and hydroxypropyl methyl cellulose acetate succinate) are readily made by reaction of polysaccharides with inexpensive propylene oxide in aqueous media.^42^ The only significant drawback to the method, for cases where such substituents would not enhance the desired performance, is the requirement for attachment of oligo(hydroxypropyl) substituents prior to bleach oxidation.

Reacting polysaccharides with reagents that contain aldehydes, ketones, or their protected analogues can be an attractive approach that is worthy of further exploration. We illustrate the approach here with esterification with 4-formylbenzoic acid.^46^ Methods described to date have been plagued with low conversion, and there is of course the issue of potential toxicity of the reagent used.^88^ Acetoacetylation of polysaccharides with diketene has attractive features, including high conversion, efficiency, and relatively mild conditions, and the acetoacetate group with its β-ketoester moiety has rich chemistry that researchers have just begun to exploit;^49,53,58,81−83,89^ as a result, it has been the topic of considerable recent research. Drawbacks of acetoacetylation for appending ketones to polysaccharides are the handling difficulties (leading to current inability to acquire it) of diketene, the most convenient acetoacetylation reagent (though alternatives like *t*-BAA exist, which requires higher reaction temperatures), and the fact that polysaccharide acetoacetates become thermally unstable at ∼100 °C. Levulinate esterification can provide ketones with improved thermal stability but is often plagued by side reactions and inefficiency in polysaccharide esterification.^62^

MREP is a highly promising new approach for adding multiple aldehyde groups to polysaccharides. Limitations to date include the relatively low DS(CHO) obtained^16^ and the fact that regioselective reactions (e.g., of appending glucosamine to polysaccharides) are so far limited to poly(uronic acids), like alginate. The concept is, however, sound; even the low DS(CHO) achieved so far does permit cross-linking and hydrogel formation,^16^ and it can be anticipated that newer methods to make MREPs will solve the issues of increasing possible DS(CHO) and enhance the breadth of regioselectivity that is achievable.

In summary, several methods have been developed to synthesize polysaccharides containing aldehyde or ketone moieties. Each method has advantages and drawbacks, but together they have created considerable application potential. There is still abundant room for the creation of new, robust, selective, efficient synthesis methods on the basis of the principles elucidated here to provide access to a broader range of stable, high-DP, targeted DS aldehyde- and ketone-substituted polysaccharides of controlled structure to enable highly challenging sustainable biomaterial applications.

## Abbreviations

DS: degree of substitution
MS: molar substitution
PS: polysaccharides
HP: hydroxypropyl
HPC: hydroxypropyl cellulose
HPD: hydroxypropyl dextran
DP: degree of polymerization
DCC: dicyclohexyl carbodiimide
DMAP: dimethyl aminopyridine
AcAc: acetoacetate
TBAA: *tert*-butyl acetoacetate
THD: 2,2,6-trimethyl-4*H*-1,3-dioxin-4-one
AGU: anhydroglucose unit
MREPs: multireducing end polysaccharides
CAA: cellulose acetoacetate
CNF: cellulose nanofibers
HA: hexylamine
ODA: octadecyl amine
HRP: horseradish peroxidase
HEMA: 2-hydroxyethyl methacrylate
MMA: methyl methacrylate
SBMA: sulfobetaine methacrylate
CA: cinnamyl aldehyde
DHP: 1,4-dihydropyridine
